# Quercetin 3-*O*-Galactoside Isolated from *Limonium tetragonum* Inhibits Melanogenesis by Regulating PKA/MITF Signaling and ERK Activation

**DOI:** 10.3390/ijms24043064

**Published:** 2023-02-04

**Authors:** Fatih Karadeniz, Jung Hwan Oh, Youngwan Seo, Jiho Yang, Hyunjung Lee, Chang-Suk Kong

**Affiliations:** 1 Marine Biotechnology Center for Pharmaceuticals and Foods, College of Medical and Life Sciences, Silla University, Busan 46958, Republic of Korea; 2 Nutritional Education, Graduate School of Education, Silla University, Busan 46958, Republic of Korea; 3 Division of Convergence on Marine Science, College of Ocean Science and Technology, Korea Maritime and Ocean University, Busan 49112, Republic of Korea; 4 Department of Food and Nutrition, College of Medical and Life Sciences, Silla University, Busan 46958, Republic of Korea

**Keywords:** cAMP, B16F10, ERK, melanogenesis, MITF, quercetin 3-*O*-galactoside, tyrosinase

## Abstract

Quercetin 3-*O*-galactoside (Q3G) is a common dietary flavanol that has been shown to possess several bioactivities, including anti-melanogenesis. However, how Q3G exerts its anti-melanogenic effect has not been studied. The current study, therefore aimed to investigate the anti-melanogenesis potential of Q3G and elucidate the underlying action mechanism in α-melanocyte-stimulating hormone (*α*-MSH)-induced hyperpigmentation model of B16F10 murine melanoma cells. Results showed that α-MSH stimulation significantly increased tyrosinase (TYR) and melanin production, which were significantly downregulated by Q3G treatment. The treatment with Q3G suppressed the transcriptional and protein expressions of melanogenesis-related enzymes TYR, tyrosinase related protein-1 (TRP-1), and TRP-2, along with the melanogenic transcription factor microphthalmia-associated transcription factor (MITF) in B16F10 cells. It was shown that Q3G downregulated MITF expression and suppressed its transcriptional activity by inhibiting the cAMP-dependent protein kinase A (PKA)-mediated activation of CREB and GSK3β. In addition, MAPK-regulated MITF activation signaling was also involved in the inhibition of melanin production by Q3G. The results suggest that the anti-melanogenic properties of Q3G rationalize further studies in vivo to confirm its action mechanism and consequent utilization as a cosmetic ingredient against hyperpigmentation.

## 1. Introduction

Melanin is a pigment responsible for the color of the skin, eyes, and hair of mammals. It is produced in specialized organelles called melanosomes, which are found in melanocytes. Melanogenesis is the name of the process that produces different types of melanin [1,2]. Although melanin protects skin from the harmful effects of ultraviolet radiation, overproduction results in unwanted complications in the skin, such as freckles, lesions, and discoloration, to name few [3].

The production of melanin, through melanogenesis, is regulated by a complex network of signaling pathways and transcriptional factors in melanosomes. A widely accepted mechanism to initiate and carry out melanin synthesis takes form around three critical enzymes: tyrosinase (TYR) and tyrosinase-related proteins 1 and 2 (TRP-1 and TRP-2) [4]. The rate-limiting enzyme of melanogenesis is TYR, which starts the melanin synthesis by hydroxylating L-tyrosine to form L-hydroxyl phenylalanine (L-DOPA). Subsequently, L-DOPA is oxidized into quinone and quinone into DOPA-chrome. The activity of TRP-2 and TRP-1, respectively, on DOPA-chrome yields the melanin. Inducing melanogenesis is achieved by the transcriptional activity of microphthalmia-associated transcription factor (MITF), which regulates the expression of TYR, TRP-1, and TRP-2 [5].

There are several external stimuli that upregulate the expression of melanogenesis-controlling enzymes via stimulated MITF expression and activity. It is now known that ultraviolet radiation, alpha-melanocyte-stimulating hormone (*α*-MSH), stem cell factors, and some other chemical agents enhance the MITF activity and, as a result, cause elevated melanin synthesis [6]. The enhancement of MITF activity is carried out by some well-studied signaling cascades, such as CREB/MITF and p38-JNK/MITF [7]. For the former, *α*-MSH elevates the cAMP levels by triggering a phosphorylation-based activation cascade, followed by cAMP-dependent protein kinase A (PKA) and cAMP response element-binding protein (CREB). Activated CREB upregulates the expression of the MITF gene by binding to the cAMP response element [8]. The increased production of MITF protein positively affects the expression of TYR and TRPs. Furthermore, mitogen-activated protein kinase (MAPK) family proteins employ a similar trend to elevate MITF expression and the consequent expression of melanogenesis enzymes. For instance, p38 and c-Jun N-terminal kinase (JNK) MAPK proteins were reported to be activated by the extracellular stimuli of melanogenesis and induce melanin synthesis via upregulated MITF expression [9,10]. On the other hand, the phosphorylation of extracellular signal regulated kinase (ERK) MAPK was reported to have an opposite effect on melanin synthesis, where activated ERK levels were translated into suppressed melanogenesis [11,12].

Naturally occurring compounds have been more favorable to consumers in the cosmetic industry, which led to a high demand for plant-based cosmeceutical agents that have skin-whitening properties. Several phytochemicals, including flavonoids, coumarins, tannins, and terpenes, have been reported to possess beneficial effects toward skin, such as antioxidant and anti-inflammatory activities [13,14]. Further research also revealed that these compounds exert skin-whitening activities via inhibition of the TYR and suppression of the melanosome uptake and distribution [13,14,15,16]. Among them, flavonoids are the one of the most common phytochemicals. They are widely found in plants as pigments and are, therefore, consumed daily through diet via fruits, grains, and vegetables. They possess a wide range of beneficial effects on human health, which is partly due to their immensely diversified chemical structures [17]. This diversity, both in chemical structure and bioactivity, made flavonoids and their derivatives target molecules for the natural pharmaceutical research [18]. Their reported bioactivities vary from antibiotic, antiviral, and antifungal, to antitumor and anti-inflammatory, which make flavonoids attract a wide range of interest as a potential drug candidate for several diseases and complications. This diversity also shows itself in the possible effects of flavonoids on melanogenesis. Several flavonoids, such as luteolin, eupafolin, kaempferol and galangin, inhibit melanogenesis [19,20,21], while some others, such as apigenin and icariin, enhance melanogenesis [22]. Quercetin belongs to a flavanol subclass of flavonoids, and its melanogenesis-inhibiting properties were reported [23,24]. However, Nagata et al. [25], and Takekoshi et al. [26,27] reported that quercetin enhanced melanogenesis in mouse melanocytes. It can be found widely in plants and is consumed daily. Quercetin 3-*O*-galactoside (Q3G) is a derivative of quercetin with a beta-D-galactosyl attached to its third carbon, and it shows similar prevalence and bioactivities as quercetin [28]. It showed antioxidant [29], hepatoproective [30], antimicrobial [31], and anti-osteoporotic [32] properties. In the current study, Q3G was isolated from *Limonium tetragonum*, a halophyte found in Asia and known for its use in folk medicine. While Yang et al. [24] and Choi and Shin [23] reported that quercetin and its glycoside derivatives inhibited melanogenesis, Mitsunaga and Yamauchi [33] and Yamauchi et al. [34] reported that quercetin derivatives also stimulated melanogenesis. A previous study reported the melanogenesis-inhibiting potential of *L. tetragonum*, and Q3G was isolated as a potential active ingredient along with another flavonol, myricetin 3-*O*-galactoside [35]. In this context, this current research aimed to elucidate the anti-melanogenic properties of Q3G in an α-MSH-induced in vitro melanogenesis model using B16F10 murine melanoma cells in regard to its effect on PKA and MAPK-mediated MITF expression.

## 2. Results and Discussion

### 2.1. Q3G Inhibits TYR and Melanin Levels

**Figure 1:**
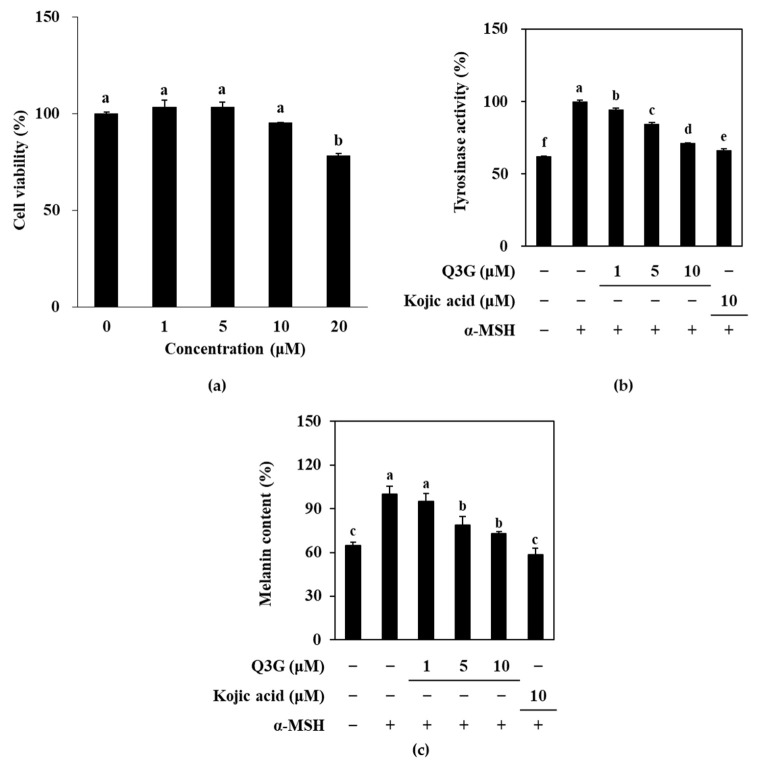
Effect of Q3G on active tyrosinase and melanin content of α-MSH-stimulated B16F10 melanoma cells. (**a**) Cells were treated with Q3G for 24 h prior to MTT assay. Viability of the cells was given as relative percentage of untreated (0 μM) group. (**b**) Tyrosinase activity was measured via ability of cell lysates to hydroxylate L-DOPA and was given as relative percentage of α-MSH-stimulated untreated group. (**c**) Total melanin contents were measured via ELISA and were given as relative percentage of α-MSH-stimulated untreated group. Kojic acid was used as positive control. ^a–f^ groups with different superscript letters are significantly different, whereas same superscript letters mean no significant difference according to Duncan’s multiple range post-hoc test (*p* < 0.05).

To assess the anti-melanogenic potential of Q3G, a melanocyte-like cell-based model was used by inducing melanogenesis in B16F10 murine melanoma cells with α-MSH-stimulation and comparing melanogenic markers with unstimulated cells and stimulated Q3G-treated cells. Initially, the effect of Q3G was investigated by measuring the intracellular active tyrosinase (TYR) and melanin content. Prior to conducting an assay of melanogenic markers in Q3G-treated B16F10 cells, non-toxic concentrations of Q3G were confirmed by an MTT assay. Results showed that the first significant decrease in viable cell amount was observed above concentrations of 10 μM (Figure 1a). Therefore, further assays were carried out with treatment doses up to 10 μM to make sure that the any effect of Q3G on melanogenesis did not arise from cytotoxicity.

Under α-MSH-stimulation, B16F10 cells were expected to produce melanin at an increased level. To measure this, first, active TYR levels were investigated according to the ability of TYR in cells to oxidize L-DOPA. As is shown in Figure 1b, α-MSH-stimulation resulted in significantly increased levels of TYR activity, which indicated that B16F10 cells overexpressed TYR compared to the unstimulated, untreated group. However, treatment with Q3G decreased the TYR activity in a dose-dependent manner, which suggests that cells treated with Q3G expressed less active TYRs. This showed that Q3G might suppress melanogenesis by inhibiting TYR expression or activity. This was further investigated through the melanin content in α-MSH-stimulated B16F10 cells. As a result of the elevated TYR levels, increased melanin production was expected. Similar to TYR levels, the melanin content of α-MSH-stimulated B16F10 cells was significantly higher compared to unstimulated cells, and Q3G treatment dose-dependently reduced melanin levels (Figure 1c). These results indicated that Q3G presence inhibited the melanin production. During the experiments, kojic acid, a reported melanogenesis inhibitor, was used as a positive control. At the same concentration, kojic acid was observed to be slightly more effective than that of Q3G. Nevertheless, the effect of Q3G on decreased TYR and melanin expression showed that Q3G might possess anti-melanogenic properties.

Some molecular docking studies revealed that some quercetin derivatives, such as quercetin-7-*O*-α-L-rhamnoside, inhibited the tyrosinase activity by interacting with the His85, His244, Thr261, and Gly281 residues of tyrosinase [36]. Similarly, Fan et al. [37] reported that quercetin bound only one site in tyrosinase and caused conformational changes where it chelated a copper with 3′,4′-dihydroxy groups, which they suggested as the reason behind inhibited tyrosinase activity. In a similar trend, the 3-*O*-galactoside derivative of the flavonoid delphinidin [38] and the 3-*O*-glucoside derivative of the flavonoid cyanidin [39] were shown to bound to tyrosinase with higher affinities that inhibited its activity. These reports further suggested that the Q3G derivative of quercetin, which is promoted to be a tyrosinase inhibitor, might act as direct tyrosinase inhibitor to reduce the melanin synthesis. However, further studies were carried out to investigate any involvement of Q3G in intracellular signaling pathways that might result in the suppressed production of TYR and related products.

### 2.2. Q3G Suppressed Expression of Melanogenic Markers

To confirm the effect of Q3G on suppressing melanin production via decreased tyrosinase levels, the expression of melanogenesis-related genes was investigated at the mRNA and protein level. Melanogenesis is regulated by the enzymatic activities of TYR, TRP-1, and TRP-2, the expressions of which are controlled by MITF [6].

**Figure 2:**
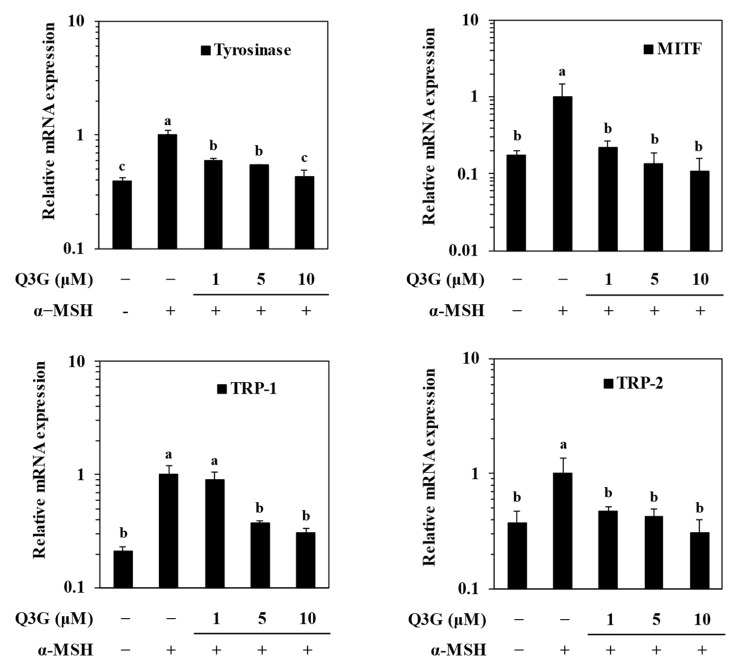
Effect of Q3G on mRNA expression levels of melanogenesis-related genes: tyrosinase, MITF, TRP-1, and TRP-2 in α-MSH-stimulated B16F10 melanoma cells. Stimulated cells were treated with or without Q3G for 24 h, and the mRNA levels were quantified with RT-qPCR. ^a–c^ groups with different superscript letters are significantly different, whereas same superscript letters mean no significant difference according to Duncan’s multiple range post-hoc test (*p* < 0.05).

**Figure 3:**
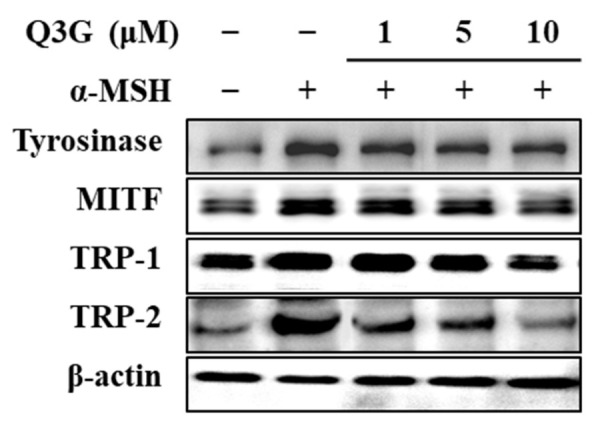
Effect of Q3G on expression of melanogenesis-related proteins, tyrosinase, MITF, TRP-1, and TRP-2 in α-MSH-stimulated B16F10 melanoma cells. Stimulated cells were treated with or without Q3G for 24 h, and the protein expression levels were quantified with western blot analysis. β-actin was used as internal loading control.

Therefore, the effect of M3G on the expression of mRNA and protein levels of MITF, TYR, TRP-1, and TRP-2 was investigated by RT-qPCR and western blotting, respectively. MITF is the transcription factor that triggers the expression of the enzymes tyrosinase, TRP-1, and TRP-2, which synthesize melanin through the hydroxylation of tyrosine. Q3G decreased the α-MSH-stimulated increase in the mRNA levels of MITF, TYR, TRP-1, and TRP-2 in a dose-dependent manner (Figure 2). Similar results were obtained from western blotting. The levels of these proteins, which were notably elevated upon α-MSH stimulation, were also suppressed dose-dependently by Q3G treatment (Figure 3). These results suggested that the anti-melanogenic properties of Q3G were expressed by downregulating the expression of melanogenic proteins, especially MITF. The suppression of MITF expression resulted in decreased levels of melanogenic enzymes and, hence, diminished melanin production [8].

### 2.3. Q3G Inhibits PKA/CREB Activation

To elucidate the mechanism by which Q3G suppressed the expression of MITF and consequently decreased the expression levels of TRY and TRPs, the PKA/MITF signaling cascade was investigated by western blotting. One of the main pathways that stimulates melanogenesis upon extracellular stimuli is through cAMP. It is a key signal transducing molecule that induces the phosphorylation of PKA. Activated PKA then subsequently activates two different signaling pathways to upregulate MITF expression: (1) activation of the CREB [8]; and (2) activation of the glycogen synthase kinase-3 β (GSK3β) [40]. Activation of the CREB is followed by binding of the CREB with CRE to upregulate MITF expression. Activation of the GSK3β, on the other hand, phosphorylates MITF to facilitate its nuclear translocation and transcriptional activity [40,41]. Therefore, firstly, the cAMP signaling pathway was investigated in α-MSH-stimulated B16F10 cells in the presence or absence of Q3G.

**Figure 4:**
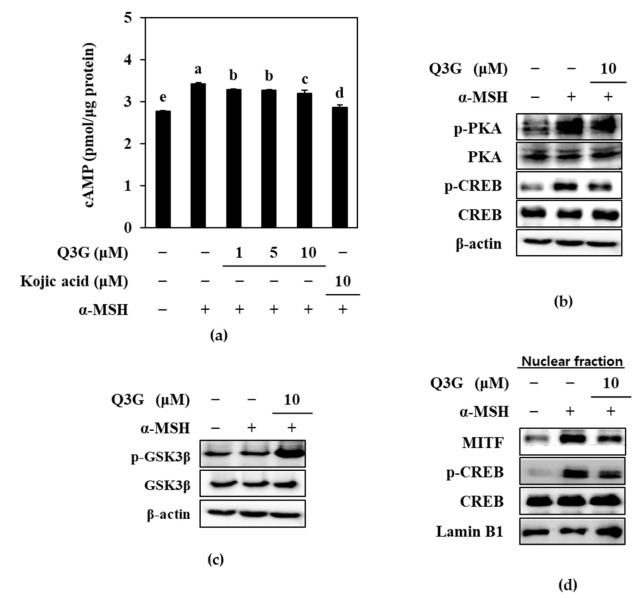
Effect of Q3G on PKA/MITF signaling pathway in α-MSH-stimulated B16F10 melanoma cells. (**a**) cAMP levels of stimulated cells were tested with ELISA after 24 h treatment with Q3G. ^a–e^ groups with different superscript letters are significantly different, whereas same superscript letters mean no significant difference according to Duncan’s multiple range post-hoc test (*p* < 0.05). (**b**) Phosphorylated (p-) and total protein levels of PKA and CREB in stimulated cells were analyzed with western blot after 24 h treatment with Q3G. β-actin was used as internal loading control. (**c**) Serine 9 phosphorylated (p-) and total protein levels of GSK3β were analyzed with western blot after 24 h treatment with Q3G. β-actin was used as internal loading control. (**d**) Nuclear levels of MITF, CREB, and phosphorylated (p-) CREB were analyzed with western blot. Lamin B1 was used as internal loading control.

The cAMP levels in treated and untreated cells were first measured via cAMP ELISA. Results confirmed that, upon α-MSH stimulation, the levels of cAMP were increased to 3.43 pmol/μg of protein in B16F10 cells compared to 2.77 pmol/μg of protein in the unstimulated group (Figure 4a). Treatment with different concentrations of Q3G decreased the cAMP levels in a dose-dependent manner. At the concentration of 10 μM, Q3G treated cells expressed 2.87 pmol/μg of cAMP protein.

Next, the phosphorylation levels of PKA, the downstream activator of cAMP signaling, and the CREB, the transcriptional factor that stimulates MITF expression, were investigated. Confirming the decrease in cAMP levels by Q3G, the results showed that Q3G treatment decreased the phosphorylation of PKA and consequently of CREB (Figure 4b). After treatment with 10 μM of Q3G, phosphorylated PKA and CREB levels were decreased, while total PKA and CREB levels were remained same.

Another pathway to induce MITF-mediated melanin synthesis through the cAMP cascade is the activation of GSK3β. Upon activation of the PKA via cAMP, PKA activates GSK3β, which subsequently stimulates MITF activity and that results in the consequent expression of TYR and TRPs [42]. However, when GSK3β is phosphorylated at serine 9, its inactivation results in decreased MITF transcriptional activity [43]. Results showed that Q3G treatment significantly increased the phosphorylation of GSK3β, which confirmed the effect of Q3G on suppressing MITF activity (Figure 4c). It was suggested that by decreasing the cAMP-mediated activation of PKA, Q3G decreased the nuclear translocation of the activated CREB and MITF.

To confirm this, the nuclear levels of total and phosphorylated CREB and MITF were investigated by western blotting. The results showed notably elevated levels of MITF protein in the nuclear fraction of α-MSH-stimulated B16F10 cells, along with higher phosphorylated CREB levels (Figure 4d) compared to the unstimulated group. Treatment with Q3G at a 10 μM concentration suppressed the nuclear MITF and phosphorylated CREB levels. These results further confirmed that Q3G decreased the MITF expression and activity in α-MSH-stimulated B16F10 cells by suppressing the cAMP-mediated activation of PKA, which led to decreased melanin production.

### 2.4. Q3G Upregulated ERK MAPK Phosphorylation

**Figure 5:**
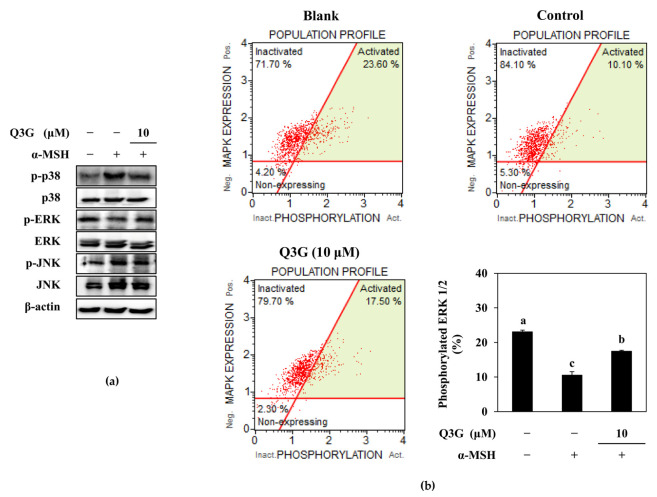
Effect of Q3G on the MAPK activation in α-MSH-stimulated B16F10 melanoma cells. (**a**) Effect of Q3G on the phosphorylated (p-) and total protein levels of p38, JNK, and ERK MAPKs was analyzed by western blot after 24 h treatment. (**b**) Activation level of ERK1/2 was measured with flow cytometry and given as percentage of activated ERK1/2 in total expressed ERK1/2. Blank: Non-stimulated untreated group, Control: α-MSH-stimulated untreated group. ^a–c^ groups with different superscript letters are significantly different, whereas same superscript letters mean no significant difference according to Duncan’s multiple range post-hoc test (*p* < 0.05).

In addition to PKA/CREB-mediated MITF regulation, studies have reported that the MAPK signaling cascade also modulates the inducement of melanogenesis, especially p38, JNK, and ERK MAPKs [9,10,11,12]. It was shown that phosphorylated ERK suppressed the MITF expression, whereas p38 and JNK phosphorylation resulted in enhanced melanogenesis by activated MITF. Therefore, the activation of p38, ERK, and JNK MAPKs were investigated in α-MSH-stimulated B16F10 cells. Results showed that melanogenesis-stimulated cells expressed increased levels of p38 and JNK phosphorylation (Figure 5a). However, phosphorylated ERK1/2 levels were notably diminished following α-MSH stimulation. Treatment with 10 μM of Q3G did not alter JNK activation. However, p38 phosphorylation was downregulated, and ERK phosphorylation was increased in the presence of Q3G. Although there are some controversial reports where ERK activation was shown to be increased in parallel with melanin synthesis [44], most of the studies reported that the phosphorylation of MITF with ERK1/2 led to its ubiquitination and consequent degradation, which resulted in suppressed melanogenesis [11,12,45]. The current results confirmed these reports where Q3G treatment elevated ERK phosphorylation. To further confirm the effect of Q3G on ERK1/2 activation, ERK activation was measured by flow cytometry. The results showed that Q3G treatment was able to increase the phosphorylated ERK1/2 levels to 17.50% of the total ERK, which were reduced to 10.10% after stimulation with α-MSH. The base ERK phosphorylation level for unstimulated untreated cells was 23.60% (Figure 5b). The notable upregulation of ERK activation and downregulation of p38 activation by Q3G in stimulated B16F10 cells suggest that modulation of the MAPK pathway was also one of the signaling cascades that Q3G intervened to reduce melanin production. The overall results suggested that the Q3G suppressed melanin synthesis via the dual mechanisms of cAMP/PKA/MITF pathway and MAPK/MITF pathway. Liu-Smith and Meyskens have summarized the molecular mechanisms of flavonoids that could affect melanin synthesis and listed an agouti signaling protein (ASIP)-mediated inhibition of melanogenesis [46]. ASIP is an antagonist of MC1R competing with α-MSH, and luteolin treatment evidently increased ASIP production in human A375 melanoma cells. They suggested that theincrease in ASIP consequently hindered the ability of α-MSH to bind to MC1R, thus subsequently stopping the activation of downstream melanogenic factors, including PKA and MAPK signaling. Considering that luteolin and Q3G structures only differ in the 3 position, where galactoside were attached to Q3G, hindering MC1R/α-MSH interaction might also be how Q3G exerted its anti-melanogenic properties via PKA and MAPK signaling pathways. However, further detailed studies are required using human cell lines and skin models to suggest any involvement of Q3G in ASIP-mediated melanogenesis inhibition.

## 3. Materials and Methods

### 3.1. Sample Material

Q3G was isolated and characterized as reported earlier [35]. Briefly, Q3G was isolated via bioactivity-guided isolation from the methanolic extract of *L. tetragonum*. ^1^H and ^13^C NMR spectra were recorded and compared with the published literature for the identification of the Q3G.

### 3.2. In Vitro Melanogenesis Model

To study melanogenesis in vitro, a murine melanoma B16F10 cell line was employed. The cell line was purchased from the Korea Cell Line Bank (Seoul, Republic of Korea). Cells were cultured in T-25 cell culture flasks (#70025, SPL Life Sciences, Pocheon, Republic of Korea) and fed with Dulbecco’s modified Eagle’s medium (DMEM, Welgene, Republic of Korea) containing 10% fetal bovine serum (FBS, Welgene) and 1% antibiotic solution (Welgene). For the assays, cells were transferred onto 6- or 96-well plates. Melanogenesis in B16F10 cells was induced by stimulating the cells with the addition of 400 nM α-MSH for 24 h. A separate group was only fed DMEM without α-MSH for comparison. During experiments, cells were kept in 37 °C incubators with controlled atmosphere containing 5% CO_2_.

### 3.3. Assessment of Cytotoxicity

To investigate any toxicity by Q3G in B16F10 cells, a common 3-(4,5-dimethylthiazol-2-yl)-2,5-diphenyl-tetra-zolium bromide (MTT) assay was carried out. Briefly, cells were transferred onto 96-well plates with a density of 3 × 10^3^ cells per well and kept for 24 h in incubators. After 24 h, cells were added with varying concentrations of Q3G for the next 24 h. Culture medium was swapped with 100 μL of MTT reagent (0.05%, *m*/*v*) afterwards, and the plates were kept in incubators for 4 h. The reaction was stopped by adding 100% DMSO to each well. Absorbance values were then measured at 540 nm. Cell viability was quantified as the absorbance value of each well normalized against a blank well containing only cells and DMSO given as a relative percentage of the untreated control.

### 3.4. Determination of Intracellular Active TYR Levels

The cellular active TYR levels were investigated by measuring the L-DOPA hydroxylation ability of Q3G treated cells. Briefly, cells were transferred to 6-well plates at a density of 5 × 10^4^ cells per well, kept in incubator for 24 h, and stimulated with α-MSH as described in Section 3.2 with or without different concentrations of Q3G. After 24 h, wells were aspirated, and cells were washed twice with PBS. Cells were then lysed via vigorous pipetting with 200 μL lysis buffer (5 mM EDTA, 0.1 M pH 6.8 sodium phosphate buffer, 1% Triton X-100, 0.1 mM PMSF). Lysates were then centrifuged at 1000× *g* for 5 min. Supernatants were tested for tyrosinase activity. The total protein contents of the supernatants were calculated by Bio-Rad protein assay solution (Bio-Rad, Hercules, CA, USA) following manufacturer’s instructions. Reaction mixtures were prepared by adding cell lysate that contained same amount of protein (20 μg) and 0.1 M sodium phosphate buffer (pH 6.8) at a ratio of 1:3. Reaction was initiated by adding 50 μL L-DOPA (0.1% *w*/*v*) to reaction tubes. Tubes were then kept at 37 °C for 1 h, and the absorbance values were measured at 490 nm. Tyrosinase activity was quantified from absorbance values and given as a relative percentage of the α-MSH-stimulated untreated control group.

### 3.5. Determination of Melanin Production

The melanin content of B16F10 cells was quantified using the cell lysates obtained in the Section 3.4. Cell lysates were washed with ice-cold 75% EtOH and air-dried at room temperature. Dried lysates were suspended in 200 μL 1N NaOH containing 1% DMSO. To facilitate the dissolution of melanin, the mixture was heated to 80–90 °C and kept at this temperature for 1 hr. Absorbance values were recorded at 405 nm. Melanin content of the cells was quantified by plotting absorbance values against a melanin standard curve, which was prepared using samples with known melanin concentrations. The standard calibration curve was prepared using purified melanin (Sigma-Aldrich, St. Louis, MO, USA).

### 3.6. Determination of cAMP Levels

The intracellular cAMP levels of B16F10 cells were analyzed using a commercial ELISA kit (cAMP Direct Immunoassay Kit; K371, Abcam, Cambridge, UK) and following the manufacturer’s directions with necessary modifications. The cAMP levels were determined in cell lysates of α-MSH stimulated B16F10 cells treated with or without Q3G prepared according to instructions enclosed with the kit. The cAMP levels were quantified with the help of a standard curve and given as picomole per microgram of protein.

### 3.7. Measuring mRNA Expression

The mRNA expression levels were analyzed by quantitative reverse transcriptase-polymerase chain reaction analysis (RT-qPCR). Cells were transferred to 6-well plates at a density of 5 × 10^4^ cells per well and kept in incubator for 24 h, after which they were stimulated with α-MSH, as described in Section 3.2, with or without different concentrations of Q3G. After 24 h, total RNA was isolated from cells using a commercial RNA extraction kit (AccuPrep^®^ Universal Bioneer, Daejeon, Republic of Korea) and following the instructions. RNase-free DNase I (Thermo Fisher Scientific, Rockford, IL, USA) treated total RNA was reverse transcripted into cDNA using CellScript All-in-One cDNA synthesis Master Mix (CellSafe, Yongin, Republic of Korea). Subsequently, quantitative polymerase chain reaction analysis was carried out with Luna Universal qPCR pre-mix (New England Biolabs, Ipswich, MA, USA) using TP800 Thermal Cycler Dice™ Real-Time System (Takara Bio, Ohtsu, Japan) according to the manufacturer’s directions. The amplification values were plotted as a relative quantity calculated at cross point and given as fold change compared to the α-MSH-stimulated untreated group. All amplification values were normalized against β-actin as the reference gene.

### 3.8. Measuring Protein Levels

The target protein levels in B16F10 cells were investigated with traditional western blotting procedure. Briefly, cells were stimulated and treated as described in Section 3.7. Q3G treated or untreated cells were then lysed by adding RIPA lysis buffer to each well and incubating for 30 min, after which a homogenous cell lysate was obtained by pipetting the lysis buffer up and down. The total protein amounts in cell lysates were quantified by Bio-Rad protein assay following the manufacturer’s instructions. Twenty micrograms of protein from each well were separated with 12% SDS-PAGE. Separated proteins were then transferred onto membranes using pre-packed Trans-Blot Turbo wet transfer system. Membranes were then blocked by being kept in 5% skim milk for 4 h and treated with primary antibodies overnight at 4 ℃. The specific protein bands were then visualized following incubation with horseradish-peroxidase-conjugated secondary antibody for 4 h at room temperature and with the help of a commercial chemiluminescence ECL assay kit (Amersham Pharmacia Biosciences). Images of the bands were taken with Davinch-Chemi CAS-400SM Imager (Davinch-K, Seoul, Republic of Korea).

### 3.9. Flow Cytometry

The phosphorylated ERK1/2 MAPK levels in B16F10 cells were analyzed by flow cytometry. Briefly, B16F10 cells were transferred to 6-well plates (5 × 10^4^ cell/well) and were stimulated and treated as described in Section 3.7. The active (phosphorylated) and inactive ERK1/2 proteins were quantified with the MUSE MAPK Activation Dual Detection Kit (MCH200104; Merck KGaA, Darmstadt, Germany) using MUSE Cell Analyzer and software (Muse Cell Soft V1.4.0.0, Merck KGaA, Darmstadt, Germany) according to the manufacturer’s instructions.

### 3.10. Statistical Analysis

The data were presented as mean ± SD (*n* = 3) where applicable. Significant differences between the means of the different treatment groups were expressed at the *p* < 0.05 level calculated by one-way analysis of variance (ANOVA) coupled with Duncan’s multiple range post-hoc test (SAS v9.1, SAS Institute, Cary, NC, USA). All groups were compared to each other, and bars were marked with different letters starting with “a”. The bars with different letters above them were significantly (*p* < 0.05) different, while bars with the same letters were not.

## 4. Conclusions

In conclusion, results showed that Q3G treatment reduced melanin synthesis in α-MSH-stimulated B16F10 melanoma cells. Stimulation induced enhanced melanogenesis, which was confirmed by increased TYR and melanin production, which were reverted by Q3G presence. Q3G was shown to decrease melanin synthesis by reducing the expression and transcriptional activity of MITF. Q3G suppressed the cAMP-mediated activation of PKA, which led to suppressed CREB and GSK3β activation. Both resulted in decreased expression and activation of the MITF. Also, Q3G treatment upregulated the ERK1/2 phosphorylation while downregulating p38 phosphorylation, which suggested involvement of the MAPK/MITF signaling pathway as well in the anti-melanogenic effect of Q3G. Therefore, Q3G is suggested to be a potential anti-melanogenic natural product, and it might be utilized as a cosmetic ingredient against hyperpigmentation.

## Data Availability

The data presented in this study are available upon request from the corresponding author.
